# Transcriptome Analysis of Apple Leaves in Response to Powdery Mildew (*Podosphaera leucotricha*) Infection

**DOI:** 10.3390/ijms20092326

**Published:** 2019-05-10

**Authors:** Xiaomin Tian, Li Zhang, Shuaishuai Feng, Zhengyang Zhao, Xiping Wang, Hua Gao

**Affiliations:** 1 State Key Laboratory of Crop Stress Biology in Arid Areas, College of Horticulture, Northwest A&F University, Yangling 712100, China; tianxiaomin0505@163.com (X.T.); zl775066779@163.com (L.Z.); 18906480991@163.com (S.F.); zhaozy@nwsuaf.edu.cn (Z.Z.); 2 Key Laboratory of Horticultural Plant Biology and Germplasm Innovation in Northwest China, Ministry of Agriculture, Northwest A&F University, Yangling 712100, China

**Keywords:** apple (*Malus × domestica* Borkh.), RNA-seq, transcription factors, fungus, signal pathway, plant hormone

## Abstract

Apple (*Malus × domestica* Borkh.) is one of the most important cultivated tree fruit crops worldwide. However, sustainable apple production is threatened by powdery mildew (PM) disease, which is caused by the obligate biotrophic fungus *Podosphaera leucotricha*. To gain insight into the molecular basis of the PM infection and disease progression, RNA-based transcriptional profiling (RNA-seq) was used to identify differentially expressed genes (DEGs) in apples following inoculation with *P. leucotricha*. Four RNA-seq libraries were constructed comprising a total of 214 Gb of high-quality sequence. 1177 DEGs (661 upregulated and 629 downregulated) have been identified according to the criteria of a ratio of infection/control fold change > 2, and a false discovery rate (FDR) < 0.001. The majority of DEGs (815) were detected 12 h after inoculation, suggesting that this is an important time point in the response of the PM infection. Gene annotation analysis revealed that DEGs were predominately associated with biological processes, phenylpropanoid biosynthesis, hormone signal transduction and plant-pathogen interactions. Genes activated by infection corresponded to transcription factors (e.g., AP2/ERF, MYB, WRKY and NAC) and synthesis of defense-related metabolites, including pathogenesis-related genes, glucosidase and dehydrin. Overall, the information obtained in this study enriches the resources available for research into the molecular-genetic mechanisms of the apple/powdery mildew interactions, and provides a theoretical basis for the development of new apple varieties with resistance to PM.

## 1. Introduction

Apple (*Malus* × *domestica* Borkh.) powdery mildew (PM), caused by the obligate biotrophic fungus, *Podosphaera leucotricha*, is one of the most prevalent fungal apple diseases, affecting almost all cultivars in all major apple-growing areas of the world [1]. Leaves and young fruits are the most susceptible organs. Infected leaves initially show white lesions on the adaxial surface and chlorotic patches on the abaxial surface. Infected leaves tend to crinkle and curl, turn brown and drop prematurely. The fungus typically overwinters in vegetative buds. In the early spring, the fungus resumes growth, and spores from infected shoots can initiate secondary infections [2]. Chronic PM infection leads to the loss of vigor and stunted growth, and can severely reduce yield [3].

Fungicides have been widely used to control PM in apple. However, frequent applications of agrochemicals are not only costly but also can have a negative effect on the environment. An improved understanding of PM-defense mechanisms and the consequent development of PM-resistant varieties would help improve the economic and environmental sustainability of apple cultivation [4].

Advances in understanding of plant-pathogen interactions have been enabled by the availability of plant and pathogen genome sequences and the development of associated bioinformatics tools. Comparative transcriptional analysis, using RNA-based sequencing (RNA-seq), is now a common approach for identifying genes that are differentially expressed between two samples [5]. This method has been widely applied in research into plant-pathogen interactions in horticultural crops, including in grape (*Vitis vinifera*) [6], pear (*Pyrus hopeiensis*) [7] and tomato (*Solanum lycopersicum*) [8]. Several such studies have also examined the interaction of apple with pathogens, including *Venturia inaequalis* [9], *Alternaria alternata* [10], *Marssonina coronaria* [11], *Valsa mali* [12] and *Pythium ultimum* [13]. These five transcriptomic datasets in *Malus × domestica* responses to fungal pathogens were analyzed. Relating to hormones, brassinosteroids were induced by fungal pathogens. Jasmonate was repressed by fungal pathogens. Most of the transcription factors were induced by fungal pathogens such as AP2-EREBP, WRKYs and ARRs. Ubiquitine-mediated degradation was upregulated by fungal pathogens. Transporters in the envelope membrane were induced by fungal pathogens. As expected, fungal pathogens repressed photosynthesis-related genes such as those involved in photosystem II. Adenylpyrophosphatase (ATPase), photorespiration and major CHO (carbohydrates) metabolism were significantly inhibited by fungal pathogens. Cell wall genes were downregulated in all the five datasets. PR-proteins (pathogenesis-related proteins) and other stress related proteins were upregulated by fungal pathogens. Thus, transcriptional analyses are playing a significant role in defining gene and protein functions and plant-pathogen interactions.

A range of apple germplasm has been evaluated for resistance to PM, involving studies of a large number of physiological responses [14], some inducing salicylic acid (SA) resistance mechanisms or silicon application [15], and others identifying and expressing resistance genes [16]. Transcriptome analysis of the PM infection in apples was presented in this paper. A high-resolution transcriptional analysis of the PM infection in the commercially valuable and highly susceptible cultivar, ‘Ruiyang’, has been presented here. The identification of gene responses to the PM infection is an important first step both for controlling PM in existing apple orchards, and for the development of new, PM-resistance cultivars.

## 2. Results

### 2.1. Statistical Analysis of RNA-seq Results from Different Time Points after PM Inoculation

To analyze the transcriptional response to the PM infection in apple, leaves were inoculated with a suspension of *P. leucotricha*, and RNA-seq analysis was performed on samples atat 0, 12, 24 and 48 h after infection (hpi). Leaves mock-inoculated with sterile water served as the controls. An overview of the sequencing and mapping results is shown in Table 1. An average of 59,609,923 reads was obtained in each sample, with a Q20 quality score ≥ 96.74%. Filtered reads were aligned with an apple genome (*Malus domestica* v1.0; 122,107 contigs) [17] obtained from the Genome databases for Rosaceae (GDR, http://www.rosaceae.org), resulting in an average mapping percentage of 79.35%. The correlation between any two of the three replicates for each treatment was > 95% (Supplementary Figure S1), indicating the high quality of the data.

### 2.2. DEG Profiles in Response to PM Infection

**Figure 1:**
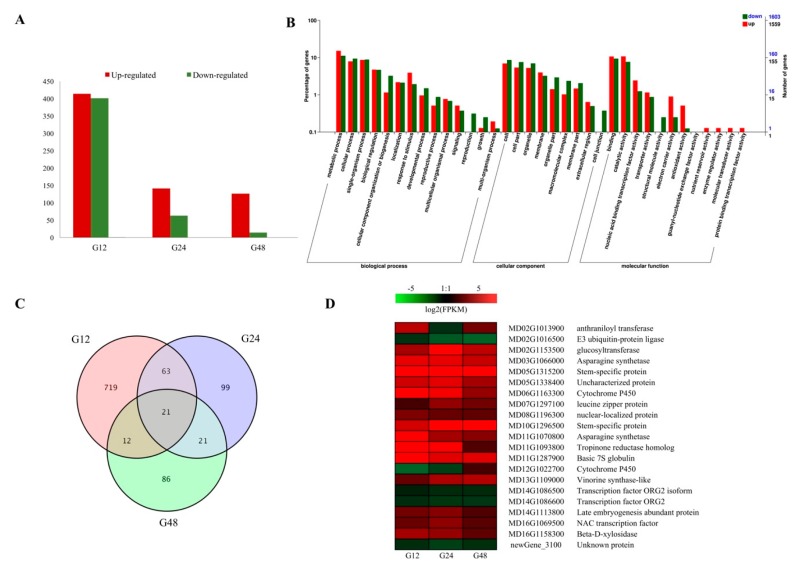
Differentially expressed genes (DEGs). (**A**) Number of DEGs between treated and control samples. DEGs are shown in red (upregulated in inoculated samples) and green (downregulated); (**B**) GO functional enrichment analysis of DEGs; (**C**) Venn diagram depicting number and overlap among DEGs from each time point; (**D**) expression profiles of selected DEGs. The maximum/minimum log_2_^FPKM^ value was set to ± 3.0 (red for upregulated, green for downregulated), each horizontal row represents a DEG while the vertical columns represent 12, 24 and 48 hpi.

Comparisons of gene expression were performed between the PM-infected and control samples: When the 24 h control and inoculated libraries were compared (T24 vs. CK24), 201 DEGs were found, with 141 being upregulated and 63 downregulated. A total of 140 DEGs were identified from the comparison between the 48h control and inoculated libraries (T48 compared to CK48), of which 126 were upregulated and 14 were downregulated in the inoculated sample compared to the control samples. Of all comparisons, the largest number of DEGs, 815, was found when the 12 h control and inoculated libraries were compared (T12 compared to CK12). Here, 414 genes were upregulated and 401 were downregulated in the inoculated sample compared to the control samples (Figure 1A). The results showed that an early response is important in the response of the PM infection. At 24 and 48 hpi, decreases in the level of fungal development, latent infection, and gene stable expression trends were apparent, based on the RNA-seq data.

Gene Ontology (GO) assignments were developed using the Blast2GO program (http://www.blast2go.com/) to generate an overview of the functional categories associated with the infection-associated DEGs. Among the annotated DEGs, 2034 were classified in the GO database, and the most significantly enriched GO terms included: ‘metabolic process’, ‘binding’, ‘catalytic activity’, ‘single-organism process’, ‘cellular process’, ‘cell part’, ‘biological regulation’ and ‘response to stimulus’ (Figure 1B).

When these results were illustrated as Venn diagram, it was clear that both unique and shared DEGs were identified between, and among, pairs (Figure 1C). By observing the DEGs throughout the infection time course, 21 DEGs were found to be upregulated in all three comparisons (12, 24 and 48 hpi). When their expression patterns were analyzed (Figure 1C), most ones were upregulated, including some transcription factors (TFs) and pathogenesis-related (PR) proteins, such as cytochrome P450, β-D-xylosidase, glucosyltransferase and asparagine synthetase.

### 2.3. Pathway Enrichment Analysis at Different Infection Stages

Kyoto Encyclopedia of Genes and Genomes (KEGG) pathways were identified according to DEGs with a corrected *p* < 0.05 at various time points. A total of 25 KEGG pathways were significantly enriched, of which 25, 24, and 18 were enriched at 12, 24 and 48 hpi, respectively, while six were enriched at all time points (Table 2). Maps with the highest DEG representation were those for ‘plant hormone signal transduction’ (KO 04075), followed by those for ‘plant-pathogen interactions’ (KO 04626), ‘phenylpropanoid biosynthesis’ (KO 00940), ‘cyanoamino acid metabolism’ (KO 00460) ‘ABC transporters’ (KO 02010) and ‘protein processing in endoplasmic reticulum’ (KO 04141). Taken together, these results suggest that the apple has evolved a range of molecular defense strategies that predominate in different infection stages, and that infection with PM leads to changes in many metabolic pathways. The process is clearly complex and is likely to be regulated by the interaction of multiple pathways, such as between multiple hormone signaling pathways.

### 2.4. DEGs Involved in Phytohormone Signaling

**Figure 2:**
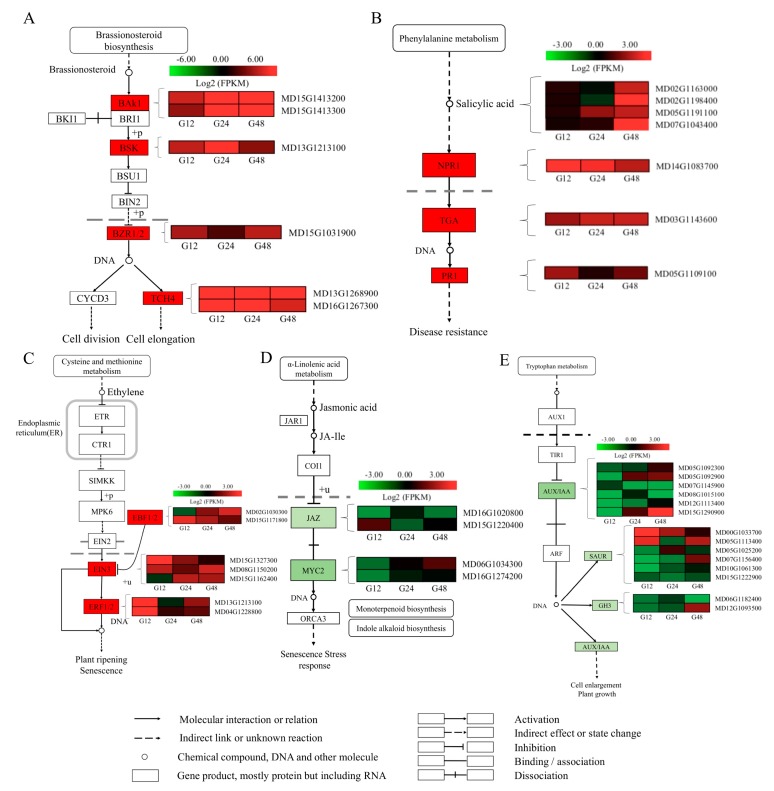
DEGs involved in phytohormone signaling transduction pathways. (**A**) Brassionsteroid (BR); (**B**) salicylic acid (SA); (**C**) ethylene (ET); (**D**) jasmonic acid (JA); (**E**) auxin (AUX). Expression values are presented as log_2_^FPKM^ value (red for upregulated, green for downregulated), and the vertical columns represent 12, 24 and 48 hpi from left to right.

Phytohormones, including salicylic acid (SA), ethylene (ET), jasmonic acid (JA), brassionosteroid (BR) and auxin (AUX), are critical regulators of plant-pathogen interactions [18]. To identify DEGs associated with hormonal response in the apple leaves infected with PM, the hormone signal transduction pathways were analyzed (Figure 2). Several genes known to be BR-responsive, including BAK1 (BRI-associated receptor kinase 1), BSK (brassionosteroid insensive 1), BZR1/2 (brassionazole-resistant transcription factor) and TCH4 (Xyloglucan endotransglucosylase, also known as Touch 4) that exhibited the same expression pattern were significantly upregulated (Figure 2A). Several DEGs involved in SA biosynthesis and signaling were differentially expressed, but only at 48 hpi. However, DEGs involved in NPR1 (non-expresser of pathogenesis-related genes), TGA (TGACG motif-binding factors) and PR1 (pathogenesis-related proteins) were significantly upregulated in the three stages (Figure 2B). Most DEGs in ethylene signaling, such as EBF 1/2 (EIN3-binding F-box protein 1/2), EIN3 (ethylene insensitive 3) and ERF 1/2 (ethylene response factor 1/2) were upregulated (Figure 2C). While most JA- and AUX-related genes were notably downregulated during infection (Figure 2D). DEGs involved in AUX signaling including MYC2 (Myelocytomatosis), AUX/IAA (auxin/indole-3-acetic acid) and Auxin-responsive GH3 (Gretchen Hagen3 genes) were differentially expressed at the early stages by 12 hpi. (Figure 2E). The related KEGG map (KO 04075) is shown in Figure S2.

### 2.5. DEGs Involved in Plant-fungal interaction

**Figure 3:**
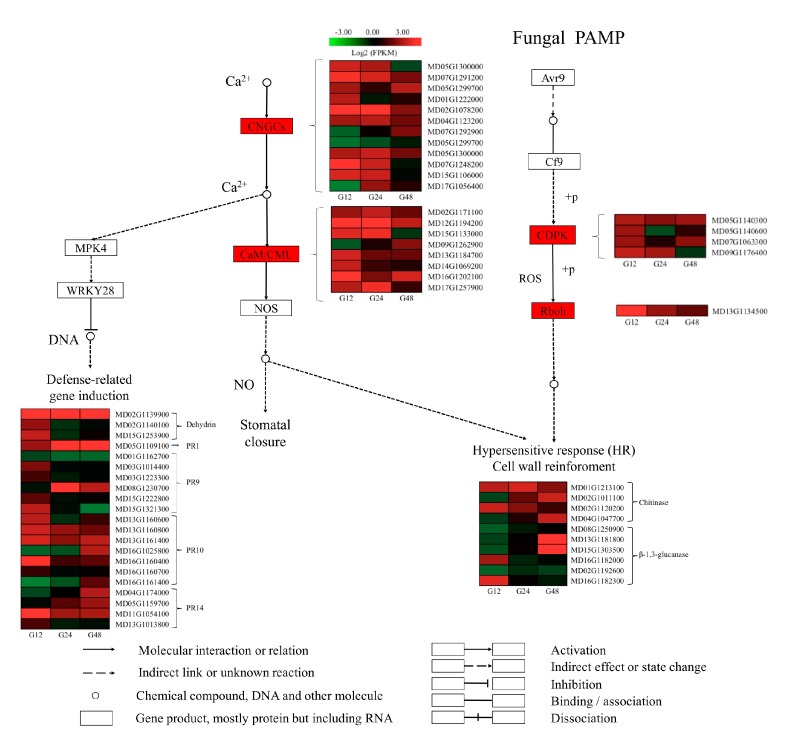
DEGs involved in plant-fungal interaction. Expression values are presented as log_2_^FPKM^ value (red for upregulated, green for downregulated), and the vertical columns represent 12, 24 and 48 hpi from left to right.

Based on the data in Table 2, ‘plant-pathogen interaction’ pathway was also analyzed, especially the fungal related pathway. The four DEGs encoding CDPK (calcium-dependent protein kinase) and Rbohs (respiratory burst oxidase homolog) were significantly upregulated at the early stages, inducing a Hypersensive response (HR) and cell wall reinforcement. Furthermore, most DEGs encoding chitinases (CHI) and glucanase-related genes were upregulated during the PM infection, however there were also some downregulated genes. Most glucosidase-like DEGs were only induced at 12 hpi, with only two upregulated by 48 hpi. Most DEGs encoding potential CNGCs (cyclic nucleotide-gated channels) were strongly up-regulated at 12 hpi and 24 hpi, while the gene expression pattern tended to stabilize at 48 hpi and DEGs encoding CaM/CMLs (Crassulacean acid metabolism/Calmodulin-Like Proteins) have similar trend. Adittionally, the defense-related genes have been induced. One of these encoding dehydrin-like was upregulated during the infection while others were upregulated only at 12 hpi. Furthermore, the expression of most PR10 (ribonuclease) and PR14 (lipid-transfer protein) genes gradually increased during the infection (Figure 3), while that of other PR genes, such as PR-9 (peroxidase) genes did not change between control and inoculated samples. The related KEGG map (KO 04626) is shown in Figure S3.

### 2.6. TFs Related to PM Responses

**Figure 4:**
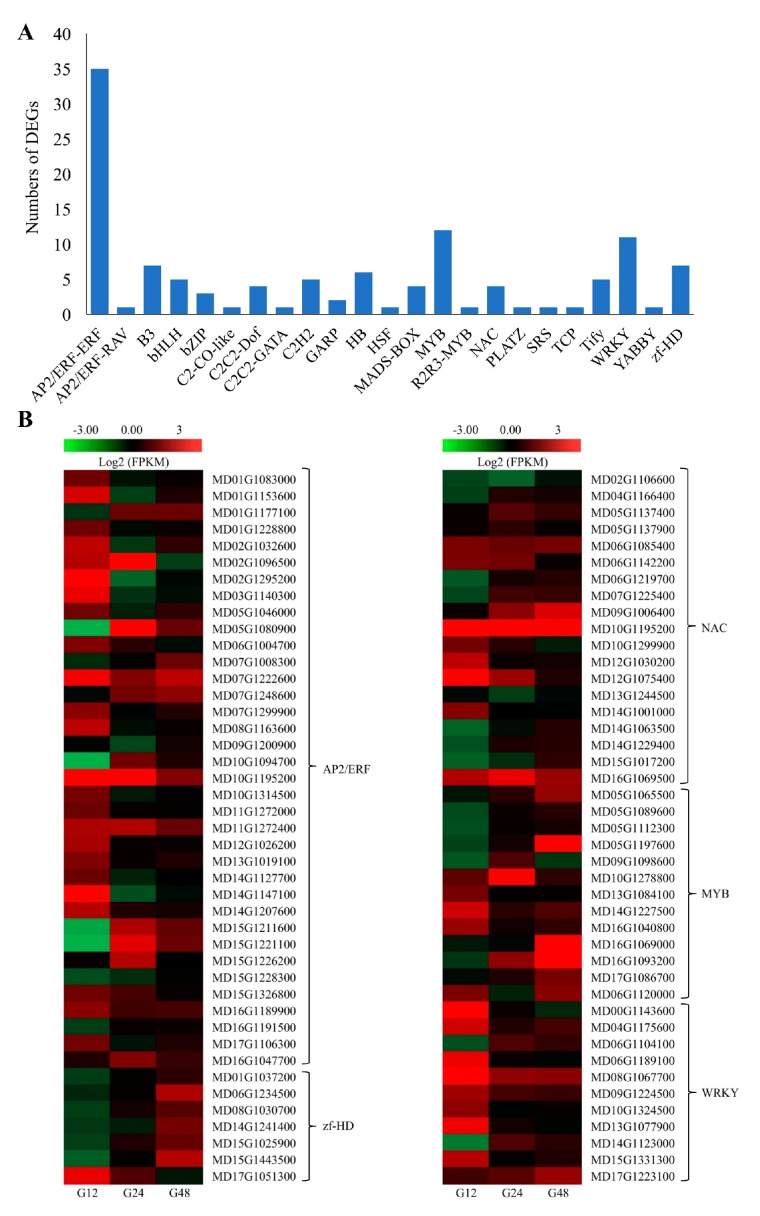
Transcription factors (TFs) in the differentially expressed genes (DEGs) sets. (**A**) Number of DEGs in different TF families; (**B**) expression profiles of genes from different TF families. The maximum/minimum log_2_^FPKM^ value was set to ± 3.0 (red for upregulated, green for downregulated), each horizontal row represents a DEG with its gene ID, and the vertical columns represent 12, 24 and 48 hpi from left to right.

TF families, including WRKY (a conserved N-terminal sequence of WRKYGQK in conjunction with a Zn finger-like motif.), NAC (N-acetyl cysteine), MYB (myeloblastosis oncogene), bZIP (basic region-leucine zipper), HD-Zip (homeodomain-leucine zipper), AP2/ERF (APETALA2/ethylene responsive factor), bHLH (basic helix-loop-helix) and ZF-TFs (zinc finger-TFs), play vital roles in regulating plant resistance mechanisms under abiotic stress [19]. In the present study, 134 TFs, from 23 families, were identified among the 1177 DEGs. The most highly represented TF class was the AP2/ERF family, with 36 DEGs. However, other classes were also well represented, including NAC, MYB, WRKY, ZF-HD, B3, HB, C2H2, TiFy, and bHLH families (Figure 4A). Interestingly, more DEGs were up- than downregulated in each TF family. The expression of the related TFs in apple induced by PM is shown Figure 4B.

### 2.7. Validation of RNA-Seq Data by Quantitative Real Time (qRT)-PCR

**Figure 5:**
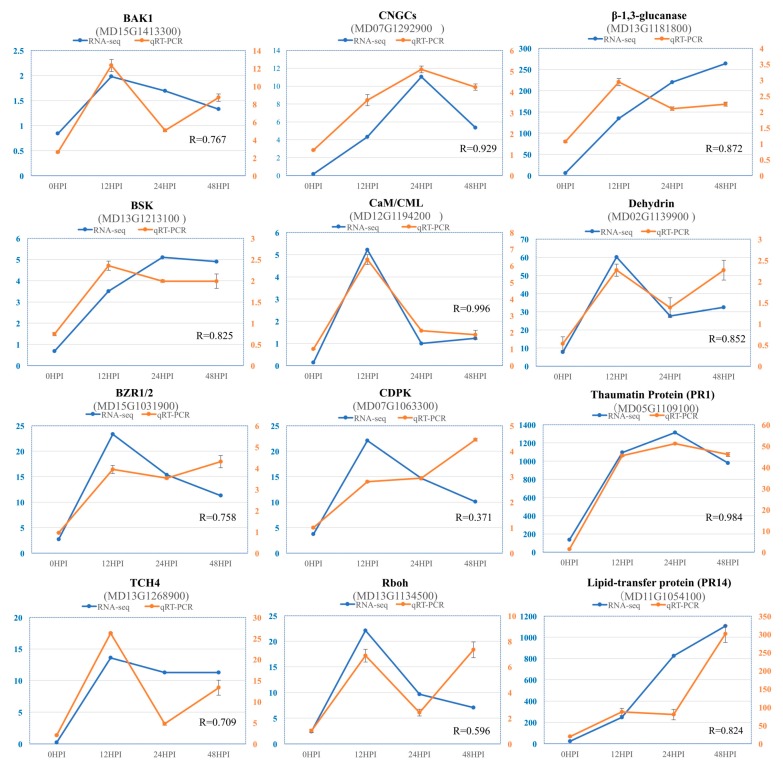
Comparison of the relative expression level change of 12 selected DEGs by RNA-seq and qRT-PCR. Left vertical axis coordinate is FPKM of RNA-Seq (blue); right vertical axis coordinate is relative expression level of qRT-PCR (red). R-values are relative coefficients between qRT-PCR and RNA-seq.

To confirm the reliability of expression levels obtained from RNA-seq transcriptome, the quantitative RT-PCR (qRT-PCR) was used to analyze expression of 12 DEGs specifically involved in BR signaling and plant-fungal interactions: BAK1 (MD15G1413300), BSK (MD13G1213100), BZK1/2 (MD15G1031900), TCH4 (MD13G1268900), CNGCs (MD07G1292900), CaM/CML (MD12G1194200), CDPK (MD07G1063300), Rboh (MD13G1134500), β-1,3-glucanase (MD13G1181800), dehydrin (MD02G1139900), PR1 (MD05G1109100) and lipid-transfer protein PR14 (MD11G1054100) (Figure 5). Both the qPT-PCR and RNA-seq analysis generally showed similar expression patterns of up and downregulation. The correlation coefficients between qRT-PCR and RNA-seq are more than 0.7 except CDPK and Rboh. Minor discrepancies regarding the expression levels, might suggest a difference in sensitivity between the two methods. These results indicate the reliability of the RNA-seq data.

### 2.8. Changes in Physiological Characteristics of Apple Leaves after Infection with PM

**Figure 6:**
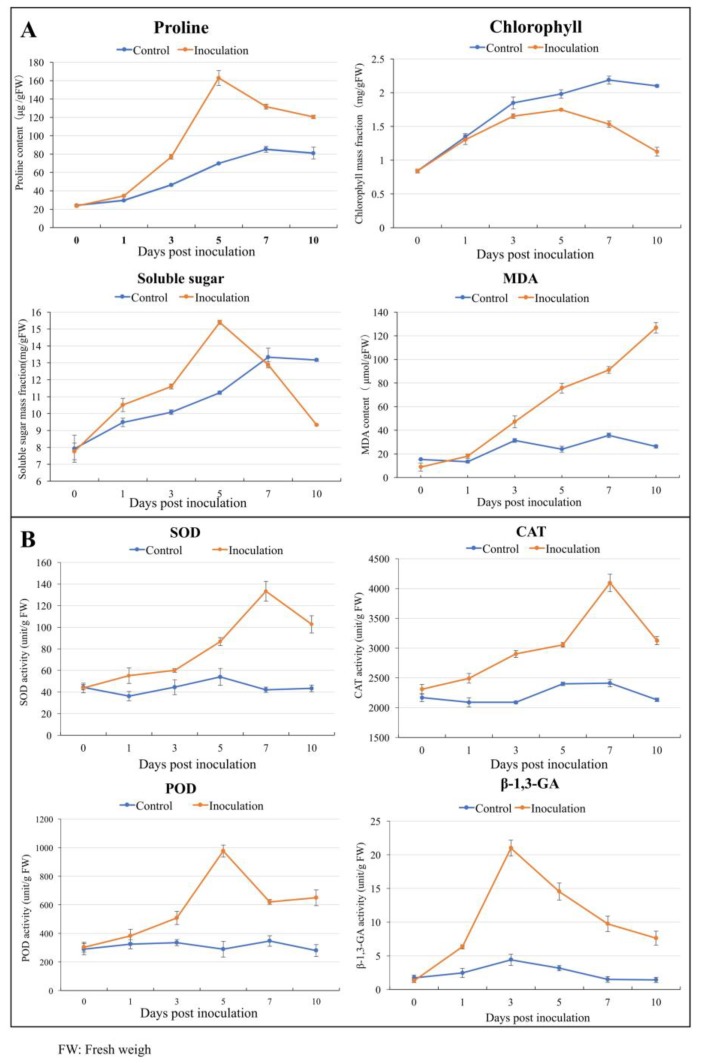
Physiological changes. (**A**) Effect of PM-infection on proline, chlorophyll, soluble sugars and malondialdehyde (MDA) content in apple leaves; (**B**) effect of PM on superoxide dismutase (SOD), catalase (CAT), peroxidase (POD) and β-1,3-glucanase (β-1,3-GA) activities in apple leaves. The leaves were incubated for various time intervals after PM inoculation. Each time point represents the mean of three measurements for each of the three biological replicates.

Many studies have used proline [20], chlorophyll [21], soluble sugars [22] and malondialdehyde (MDA) [23] content as parameters to evaluate plant tolerance to disease. These indicators in the leaves after the PM infection were measured (Figure 6A). In the control leaves, the level of proline increased consistently from one day post inoculation (dpi) to 10 dpi. In contrast, infection with PM was associated with a sharp and transient burst in proline content and the level increased from 1 dpi and reached a maximum at 5 dpi, had 235%, 155% and 150% higher proline content, respectively, at 5, 6 and 7 dpi, compared to the control (Figure 6A). Interestingly, the chlorophyll content in the control leaves increased substantially from 1 dpi to 10 dpi and remained at significantly higher levels than in the PM infected leaves. It increased from 1 dpi, reached a maximum level at 5 dpi and then declined until the end of the experiment, and the level in the control was 2-fold higher than the treatment at 10 dpi (Figure 6A). From 1 dpi to 5 dpi, the soluble sugar content increased in both the control and PM infected leaves, but the growth rate of the treated leaves was faster than that of the control. The soluble sugar content in the treated leaves declined sharply to the control level at 5 dpi. Interestingly, the soluble sugar content in inoculation leaves was 1.5-fold higher than that in the control at 5 dpi, while the soluble sugar content in the control was 1.5-fold higher than that in the inoculation at 10 dpi (Figure 6A). As shown in Figure 6A, the MDA contents in PM treated leaves increased rapidly and reached the highest value at 10 dpi, which was approximately 6-fold higher than for the control, which did not show any significant differences over the time course (Figure 6A). The expression of genes involved in the metabolism of proline, sugars, and chlorophyll were also analyzed in Figure S6.

Under abiotic stress, plant disease resistance is largely dependent on the initiation of the antioxidant enzyme system [24]. The β-1,3-glucanases participate in plant defense against fungal pathogens [25]. We measured peroxidase (POD, EC 1.11.1.7), superoxide dismutase (SOD, EC 1.15.1.1), catalase (CAT, EC 1.11.1.6), and β-1,3-glucanase (β-1,3-GA, EC 3.2.1.73) activities in PM infected leaves (Figure 6B). In leaves from the control plants, activities of the four enzymes exhibited no obvious changes during the infection period. In contrast, in infected leaves, enzyme activities were significantly (*p* < 0.05) higher than the control with varying patterns. For example, SOD and CAT activities reached their maximum levels at 7 dpi (more than 3-fold higher than the control) (Figure 6B). POD activity increased and reached a maximum level at 5 dpi, which was approximately 3-fold higher than that in the control leaves (Figure 6B). Compared with the controls, the β-1,3-GA activity in infected leaves was always significantly different. Three days after inoculation, we observed that the β-1,3-GA activity reached the maximum value (more than 4-fold higher than the control) and then decreased sharply, but remained significantly higher than that of the controls (Figure 6B).

## 3. Discussion

PM is one of the most pervasive and important fungal diseases of apple, yet there have been few previous studies of the molecular response of the apple host to the pathogen that might provide information about apple disease resistance mechanisms. RNA-based sequencing (RNA-seq), has been widely applied in research into several fungal apple pathogens, including (*Venturia inaequalis*, *Alternaria alternate*, *Marssonina coronaria*, *Valsa mali*, and *Pythium ultimum*), but none applied in powdery mildew of apple. In this study, the gene expression between apple leaves subjected to fungal infection and those grown without pathogens were compared, a total of 1177 DEGs were identified.

DEGs for KEGG pathways enrichment were analyzed. The most significant enrichment occurred in ‘hormone signal transduction’; the phytohormones, including the BR, SA, ET, AUX and JA pathways, which are known to play a role in plant-pathogen interactions [26]. Various research has pointed to a complex positive role of BRs in innate immunity [27]. We found that several DEGs associated with BR signaling in this research were strongly upregulated in response to the PM infection, suggesting that BRs could participate in regulating the response to PM in apple. SA is thought to mediate defense signaling in response to biotrophic and hemibiotrophic pathogens, while JA and ET are associated with defense responses to necrotrophs [28]. Our results suggest that SA might also play a role in local immunity against necrotrophic pathogens. Finally, another regulator of plant-microbe interactions, AUX, is known to behave predominantly as a crucial regulator of plant-microbe interactions [29]. Our expression data also suggested that AUX and JA signaling were strongly repressed by fungal infection, which is consistent with previous studies.

The number of DEGs associated with the ‘plant-pathogen interactions’ pathway was also higher in infected leaves compared with control leaves. We analyzed this subset of DEGs, especially the fungal related pathway. Several DEGs encoding CDPK and Rbohs were identified, and were found to be significantly upregulated at the early stages of infection. During infection, ROS accumulate, resulting in the hypersensitive response (HR) and cell wall reinforcement. When a plant is infected by a pathogen, a series of defense responses is activated, including the synthesis of PR proteins. Dehydrins (DHNs) are another family of proteins that protect cells from damages caused by a variety of abiotic stresses [30]. One of the DEGs (MD02G1139900) encoding dehydrin this was upregulated during the infection. This suggests the involvement of dehydrin in resistance to powdery mildew. Furthermore, β-1,3-glucanase is a PR protein that functions in the defense against fungal infection by hydrolyzing β-1,3-glucan, a fungal cell wall polyer, thus inhibiting fungal growth [31,32]. The expression of this β-1,3-glucanase related gene was verified by qRT-PCR. In addition, the activity of β-1,3-glucanase in apple leaves infected with PM was always significantly higher than that of the control group in the determination of physiological parameters, providing an evidence of the importance of β-1,3-glucanase. Many chitinases (CHI) related genes were also upregulated during PM infection. The accumulation of PR proteins is usually associated with systemic acquired resistance to a wide range of pathogens. The expression of most PR10 and PR14 genes gradually increased during the infection. Two of them have been examined by qRT-PCR, and found that at least the PR14 gene was significantly upregulated.

Many studies have used Pro [21], chlorophyll [22], soluble sugars [23] and MDA [24] content as parameters to evaluate plant tolerance to disease. These indicators in the apple leaves after the PM infection were also measured. As an osmotin, Pro and soluble sugars are important plant defense tools against abiotic stresses [21]. Compared with the control, the level of Pro and soluble sugars increased consistently during the PM infection. In order to explore the changes of the ROS enzyme activity after PM-infection in apple leaves, some indicator tests were conducted. In this study, PM treatment markedly increased the activities of POD, SOD and CAT. These are antioxidant enzymes that regulate the metabolism of ROS [33]. POD is a prerequisite for lignin synthesis and is therefore important for cell wall reinforcement. POD may also alter the antioxidant capacity of the plant, thereby increasing tolerance to fungi [33]. SOD is believed to play a crucial role in antioxidant defense as it catalyzes the dismutation of O_2_ into H_2_O_2_, which is then removed through the action of CAT and/or POD [34]. In parallel, our analysis indicated that the capacity of the tissue to scavenge excess ROS increased, and lipid peroxidation (MDA content) remained at a lower level in control leaves than that in infected leaves. These results suggest that the high levels of antioxidant enzymes associated with PM are important for reducing oxidative stress, and this is in agreement with previous reports that increased POD, CAT and SOD activities that correlate with plant disease resistance [35].

In this pathosystem, PM infection was associated with the altered regulation of TFs belonging to diverse families, including WRKY [36], zinc finger protein [37], bZIP [38] and NAC [39]. All of these TFs families are known to function in plant defense response. There is also growing evidence that AP2/ERF genes affect defense responses to fungal pathogens and environmental stimuli, and that they regulate responses through biosynthesis, perception and signal transduction, causing major changes in gene expression [40]. Here, 36 AP2/ERF genes were identified exhibiting differential expression out of a total of 1177 DEGs.

Two pathways (hormone signal transduction and plant-pathogen interactions) with the largest numbers of DEGs have been analyzed in this study. One notable outcome was that the KEGG pathway ‘phenylpropanoid biosynthesis’ (KO 00940) was also significantly enriched (Figure S4). Phenylpropanoid compounds are natural products derived from cinnamic acid, which is formed from phenylalanine via deamination by the action of phenylalanine ammonia-lyase (PAL). The phenylpropanoid pathway provides precursors for the formation of monolignols/lignin, coumarins, benzoic acids, stilbenes, and flavonoids/isoflavonoids, as well as stilbenes, a small family of secondary metabolites that have antifungal activity [41]. Some studies have demonstrated that cytochrome P450s (CYPs) can enhance plant disease tolerance by synthesizing antimicrobial compounds [42,43]. CYPs were also analyzed, as shown in this study (Figure S5). Other compounds synthesized by CYPs inhibit the growth of pathogenic microbes or take part in hormone signaling pathways to activate plant disease tolerance [44]. In this current study, most CYPs genes were upregulated during the PM infection. Therefore, CYPs might be involved in apple defense responses. These results provide some possible directions for our further research.

**Figure 7:**
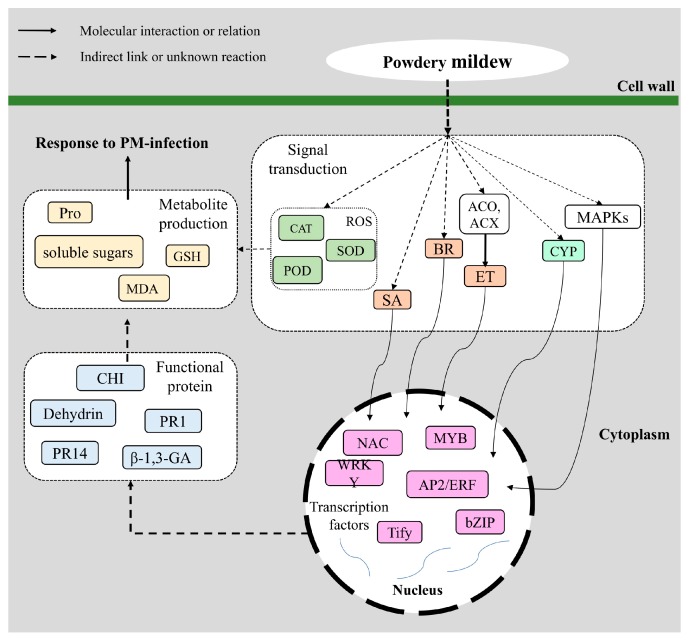
Molecular network underlying the defense response to powdery mildew (PM) in apple leaves.

Based on the results and those of previous studies, a possible molecular network underlying the defense response in apple leaves to the PM infection was diagrammed (Figure 7). The process can be divided into four stages: Signal sensing and transduction, transcriptional regulation, the synthesis of downstream functional proteins and the production of metabolite. Briefly, when apple leaves are exposed to PM, the pathogen attacks the cell wall and simultaneously triggers relevant transduction pathways, such as ROS burst and protein kinase activity. Subsequently, these cellular activities lead to the biosynthesis and signaling of plant hormones including ET, JA and JAZ. These pathways further activate secondary and tertiary regulatory networks, including TFs. TFs then activate or inhibit the expression of downstream vital functional genes, such as PRs, β-1,3-GA and CHI, to produce a response to the PM infection. The synthesis of some metabolites changes some physiological indexes of plants. It is conceivable that the genotype-specific variations, such as the timing, intensity and duration of defense reactions may lead to different dynamics of defense response.

Overall, this study represents a first step to understand the molecular mechanisms involved in resistance to PM. The transcriptome data generated here will help guide further research to develop novel strategies for disease management in apple.

## 4. Materials and Methods

### 4.1. Plant Materials, Fungal Collection and Inoculation

*Malus* × *domestica* Borkh. ‘Ruiyang’ is a newly developed late-ripening Chinese apple that exhibits sensitivity to PM. Approximately, 150 two-year-old potted apple seedlings were maintained in open-air conditions at Northwest A&F University, Yangling, China (lat. 34° 16’ 56.24”N, long. 108° 4’ 27.95” E).

Isolates of *Erysiphe necator* were collected from naturally infected plants in a nearby experimental field. For inoculation, freshly prepared spore suspensions were sprayed evenly on the abaxial side of pre-selected young leaves, which were then bagged for 24 h to maintain humidity. As a control, healthy young leaves were sprayed with sterile water. The leaves were collected at 0 h, 12 h, 24 h and 48 h after inoculation. Leaf samples were frozen in liquid nitrogen and stored at −80 °C prior to RNA extraction. Each stage contained nine parallel leaves from three apple trees that represented three biological replicates.

### 4.2. RNA Quantification and Qualification

Total RNA was extracted using the E.Z.N.A. Plant RNA Kit (Omega Bio-tek, Norcross, GA, USA). Equal quantities of RNA from three biological replicates at each stage were pooled to construct a complementary cDNA library. RNA concentration and quality was measured using a NanoDrop 2000 spectrophotometer (Thermo Fisher Scientific, Waltham, Massachusetts, USA) and RNA integrity was assessed using the RNA Nano 6000 Assay Kit and the Agilent Bioanalyzer 2100 system (Agilent Technologies, Palo Alto, CA, USA). Equal quantities of RNA from three biological replicates at each stage were pooled to construct each library. First-strand cDNA was synthesized using the PrimeScriptTM RTase 1st Strand cDNA Synthesis Kit (TaKaRa Biotechnology, Dalian, China). cDNA library construction was performed as described below and sequencing was performed at Biomarker Technologies Co, LTD (Beijing, China) using an Illumina HiSeq2500 platform (Illumina, San Diego, CA, USA).

### 4.3. Library Preparation

A total of 1 μg RNA per sample was used as input material for the RNA sample preparations. Sequencing libraries were generated using the NEB Next UltraTM RNA Library Prep Kit for Illumina (NEB, Ipswich, MA, USA) following the manufacturer’s recommendations. In order to select cDNA fragments of 240 bases in length, the library fragments were purified with the AMPure XP system (Beckman Coulter, Beverly, Brea, FA, USA). The size-selected, adaptor-ligated cDNA was then incubated with 3 μL USER Enzyme (NEB, USA) at 37 °C for 15 min followed by 5 min at 95 °C. PCR was then performed with the Phusion High-Fidelity DNA polymerase, Universal PCR primers and Index (X) Primer. PCR products were then purified (Beckman Coulter, Brea, CA, USA) and the library quality was assessed on the Agilent Bioanalyzer 2100 system (Agilent Technologies, Palo Alto, CA, USA)

Clustering of the index-coded samples was performed using a cBot Cluster Generation System with a TruSeq PE Cluster Kit v4-cBot-HS (Illumina, USA), according to the manufacturer’s instructions. After cluster generation, the library preparations were sequenced at Biomarker Technologies Co., LTD (Beijing, China) using an Illumina HiSeq2500 platform to generate paired-end reads.

### 4.4. RNA-Seq DataAnalysis

Raw data (raw reads) in a FASTQ format were initially processed through in-house Perlscripts. This step removed containing adapters, reads containing and poly(N), as well as low-quality reads from the raw data. At the same time, the Q20 and Q30 values, the GC-content and the sequence duplication level of the filtered data were calculated. All the downstream analyses were based on these filtered high-quality reads. Reads were mapped to the reference genome sequence using Tophat2 tools [45]. Gene function was annotated based on the following databases: NR (NCBI non-redundant protein sequences); NT (NCBI non-redundant nucleotide sequences); Pfam (Protein family) [46]; KOG/COG (Clusters of Orthologous Groups of proteins) [47]; Swiss-Prot (A manually annotated and reviewed protein sequence database) [48]; KO (KEGG Ortholog database) [49]; GO (Gene Ontology) [50].

### 4.5. Differential Expression Analysis

Gene expression levels were quantified as fragments per kilobase of transcript per million fragments mapped (FPKM). The formula used is as follows:

$$FPKM=\frac{cDNA\;Fragment}{Mapped\;Fragment(Million)\times Transcript\;Length(kb)}$$

For the samples with biological replicates, the differential expression analysis of two groups was performed using the DESeq R package (1.10.1) [51]. The resulting *p* values were adjusted using the Benjamini and Hochberg’s approach for controlling the false discovery rate (FDR) [52]. Genes with an adjusted *p*-value < 0.05 based on DESeq were considered to be differentially expressed. Genes with an adjusted *p*-value < 0.05 found by DESeq were assigned as differentially expressed.

### 4.6. GO and KEGG Pathway Enrichment Analysis

GO enrichment analysis of the DEGs was performed using the GOseq R packages based Wallenius non-central hyper-geometric distribution, which can adjust for gene length bias in the DEGs. KEGG was used to map sequences to pathways (http://www.genome.jp/kegg/), and the KOBAS [53] software was used to test the statistical enrichment of differentially expressed genes identified in the KEGG pathways.

### 4.7. qRT-PCR Validation and Analysis

Gene-specific primers for the qRT-PCR analysis were designed using the Beacon Designer 7.0 software (Premier Biosoft International, Palo Alto, CA, USA). An apple tubulin gene was selected as the constitutively expressed reference. All primers used are listed in Table S1. The qRT-PCR analysis was performed with SYBR Green (TaKaRa Biotechnology) with a Step OnePlus Real-Time PCR System (Applied Biosystems, Foster, CA, USA). Cycling parameters were: 95 °C for 30 s and 42 cycles of 95 °C for 5 s and 60 °C for 30 s. Each reaction was performed in triplicate for each of the three biological replicates. Relative expression levels of the selected genes were calculated using the relative 2^−ΔCT^ method [54]. The results represented in this case represent mean standard deviations for the three experimental replicates.

### 4.8. Effects of PM on Physiological Indexes

Fresh leaves from similar positions were collected at 0, 1, 3, 5, 7 and 10 days post-inoculation (dpi) and the experiment was conducted twice with three replications. Leaves were frozen in liquid nitrogen and stored at −80 °C until further use.

The content of proline was determined by the acid ninhydrin colorimetric method. Apple leaves were homogenized in 3% sulfosalicylic acid to extract free proline content. Under acidic conditions with heating, free proline and ninhydrin were heated to form stable red condensation compounds with an absorption peak at 520 nm. The red product produced by the full extraction reaction of toluene was used for the colorimetric determination, and the content of free proline was calculated from the standard curve [55]. The method described by Zude-Sasse et al. was adapted to determine the chlorophyll content. Leaves (0.2 g) were placed in 95% ethanol, and the sample was maintained in the dark for 12 h before increasing the volume to 25 mL. Absorbance was then measured at 665, 649 and 470 nm on a spectrophotometer (Hitachi, Tokyo, Japan) [56]. The content of soluble sugars was determined by the anthrone colorimetry. In the presence of 80% concentrated sulfuric acid, the sugar can be dehydrated to form furfural, which can be reacted with anthrone to form blue-green compounds. The absorbance was measured at 620 nm [57]. MDA levels were measured using the thio-barbituric acid (TBA) reaction method. Approximately 0.2 g of leaf was approximately 5 mL 5% TCA. After centrifugation, 2 mL supernatant was removed and mixed with 2 mL 0.67% TBA, and then the mixture was boiled 15 min in water bath and the supernatant was cooled and centrifuged again. The absorbance of the supernatant was measured at 450 and 532 nm and subtracted from the absorbance at 600 nm [58].

### 4.9. Effects of PM on the Accumulation of Defense-Related Enzyme

Proteins were extracted for measurements of peroxidase (POD), superoxide dismutase (SOD), catalase (CAT), and β-1,3-glucanase (β-1,3-GA) activities [58]. POD activity was estimated using the guaiacol method [58]. The reaction mixture contained 100 mL 0.2% guaiacol, 90 mL PBS and 100 mL 0.3% H_2_O_2_; 2.9 mL of the reaction solution was added with a 100 μL crude enzyme solution. Change in the absorbance at 470 nm wavelength was measured every 30 s for 3 min and BPS was used as a control. POD activity was determined using guaiacol as a substrate. A mixture of 100 mL 0.2% guaiacol, 90 mL PBS and 100 mL 0.3% H2O2 was used as the reaction solution. The reaction solution (2.9 mL) was added with a 100 μL crude enzyme solution. The absorbance was measured at 470 nm; PBS was used as the control. SOD and CAT activity was measured as described by Yong et al. [59]. In the presence of H_2_O_2_, peroxidase can oxidize guaiacol to produce brown-red products. The absorption value was measured at 240 nm [59]. For the β-1,3-glucanase activity assay, a crude enzyme solution was prepared as described by Ippolito et al. [60]. An enzyme activity unit was defined as the amount of enzyme that catalyzes the production of 1g glucose per minute per gram of fresh tissue.

## Figures and Tables

**Table 1:** Number of high-quality, filtered rate. CK, control; T, inoculated with *P. leucotricha*.

Samples	Time Points	Total Reads	Q20 Percentage (%)	Q30 Percentage (%)	Mapped Reads	Mapping Rate (%)
CK01	0 hpi	60,137,610	97.4	93.65	48,293,530	80.31
CK02	0 hpi	61,254,152	97.5	93.83	49,772,328	81.26
CK03	0 hpi	58,546,454	97.46	93.77	47,250,937	80.71
CK121	12 hpi	48,161,690	97.55	94.05	37,977,254	78.85
CK122	12 hpi	60,404,448	96.95	92.80	47,385,808	78.85
CK123	12 hpi	62,016,814	97.29	93.41	49,706,083	80.15
CK241	24 hpi	54,165,314	96.78	92.46	42,586,645	78.62
CK242	24 hpi	64,515,854	96.86	92.67	51,035,900	79.11
CK243	24 hpi	70,503,200	96.76	92.47	55,360,754	78.52
CK481	48 hpi	64,182,946	97.06	92.98	51,398,939	80.08
CK482	48 hpi	56,164,490	96.91	92.69	44,903,336	79.95
CK483	48 hpi	56,864,248	96.76	92.42	44,919,501	78.99
T01	0 hpi	68,815,498	97.41	93.67	56,225,369	78.99
T02	0 hpi	60,247,576	97.12	93.17	48,129,005	78.99
T03	0 hpi	60,534,844	97.13	93.16	48,533,584	78.99
T121	12 hpi	55,781,200	96.74	92.89	44,030,266	78.99
T122	12 hpi	68,568,070	97.03	92.94	54,673,257	78.99
T123	12 hpi	53,809,766	97.21	93.29	43,094,498	80.09
T241	24 hpi	66,547,810	96.74	92.47	52,184,112	78.42
T242	24 hpi	55,181,416	97.03	92.96	43,627,644	79.06
T243	24 hpi	43,835,550	96.86	92.63	34,502,703	78.71
T481	48 hpi	56,609,920	96.89	92.69	45,122,462	79.71
T482	48 hpi	52,151,048	96.9	92.77	40,989,977	78.60
T483	48 hpi	71,638,240	96.81	92.55	56,853,084	79.36
Average		59,609,923	97.05	93.02	47,439,874	79.35

Q20 percentage: The ratio of nucleotides with quality value ≥ 20; Q30 percentage: The ratio of nucleotides with quality value ≥ 30; Mapping rate: The percentage of mapped reads of the total high quality, filtered reads.

**Table 2:** Results of Kyoto Encyclopedia of Genes and Genomes (KEGG) pathway enrichment analysis.

Pathway Name	Number of Genes with Pathway Annotation				
	No.	Pathway ID	G12	G24	G48
Plant hormone signal transduction	P1	KO 04075	15	10	5
Plant-pathogen interaction	P2	KO 04626	11	8	1
Phenylpropanoid biosynthesis	P3	KO 00940	10	8	5
Cyanoamino acid metabolism	P4	KO 00460	7	8	3
ABC transporters	P5	KO 02010	6	7	1
Protein processing in endoplasmic reticulum	P6	KO 04141	5	7	1
Starch and sucrose metabolism	P7	KO 00500	4	5	3
Cysteine and methionine metabolism	P8	KO 00270	4	4	7
Phagosome	P9	KO 04145	4	3	1
Phenylalanine metabolism	P10	KO 00360	3	3	2
Endocytosis	P11	KO 04144	3	3	1
Stilbenoid, diarylheptanoid and gingerol biosynthesis	P12	KO 00945	3	3	1
alpha-Linolenic acid metabolism	P13	KO 00592	3	3	0
Ubiquitin mediated proteolysis	P14	KO 04120	3	3	0
DNA replication	P15	KO 03030	3	3	0
Lysine degradation	P16	KO 00310	3	3	0
Linoleic acid metabolism	P17	KO 00591	3	3	0
Glutathione metabolism	P18	KO 00480	2	3	1
Sesquiterpenoid and triterpenoid biosynthesis	P19	KO 00909	2	1	1
Biosynthesis of amino acids	P20	KO 01230	2	0	1
Glycine, serine and threonine metabolism	P21	KO 00260	2	1	1
Arginine and proline metabolism	P22	KO 00330	2	1	1
Steroid biosynthesis	P23	KO 00100	2	1	3
Carbon metabolism	P24	KO 01200	2	2	0
SNARE interactions in vesicular transport	P25	KO 04130	2	1	0

G12, G24, G48 represent the group of DEGs at 12 h, 24 h, 48 h, respectively.

## Supplementary Materials

Supplementary materials can be found at https://www.mdpi.com/1422-0067/20/9/2326/s1.

## Abbreviations

PM	Powdery mildew
DEG	Differentially expressed gene
HPI	Hours post inoculation
DPI	Days post inoculation
PCR	Polymerase chain reaction
qRT-PCR	Quantitative real time PCR
GO	Gene Ontology
KEGG	Kyoto Encyclopedia of Genes and Genomes
KO	KEGG Orthology
ZF-TFs	Zinc finger-transcription factors
PR5	Thaumatin protein
PR10	Ribonucleases
PR14	Lipid-transfer protein
CYPs	Cytochrome P450 genes
ET	Ethylene
SA	Salicylic acid
JA	Jasmonic acid
GA	Gibberellin
AUX	Auxin
BR	brassionosteroid
NBT	Nitro blue tetrazolium
DAB	Diaminobenzidine
Pro	Proline
MDA	Malondialdehyde
POD	Peroxidase
SOD	Superoxide dismutase
CAT	Catalase
β-1,3-GA	β-1,3-glucanase
CHI	Chitinase
PAL	Phenylalanine ammonia-lyase
ROS	Reactive oxygen species
