# Supplementary material for: Transcriptome Analysis of Apple Leaves in Response to Powdery Mildew (Podosphaera leucotricha) Infection
Source: Int J Mol Sci. 2019 May 10;20(9):2326. doi: 10.3390/ijms20092326 (PMC6539105; doi:10.3390/ijms20092326)
Supplement: Supplementary file 1 [file ijms-20-02326-s001.zip › All supplementary files/Table S1 .pdf]

Table S1 Genes and primer pairs used for quantitative real-time PCR

Gene function annotation	Gene ID	Forward primer (5'→3')	Reverse primer (5'→3')	
BAK1	MD15G1413300	AACCCAAGACGGGGAACTAC	CCGGACGACCTCTTAAACAA	172
BSK	MD13G1213100	TTTTGATTCCCCAAATTCCA	TGCAAAAACAGAGACGCTTG	186
BZK1/2	MD15G1031900	CGATACTTCCACCGCAGATT	CGTGAGCTCCAAGTCATCAA	204
TCH4	MD13G1268900	TGGTGGTCTTCGGGTTCTAC	GTTGCTGGCAGTGCATTCTA	191
CNGCs	MD07G1292900	ATGTGGCTGCAAATACCACA	CACATATTCAGCGCCTCTCA	226
CaM/CML	MD12G1194200	GCCTCAACTTCGACGACTTC	AGCACCACCTGCAACTCTTT	232
CDPK	MD07G1063300	GACAGGGGCAGTTTGGAATA	AGAGCTCACCTCCTGCACAT	239
Rboh	MD13G1134500	CTCGAGCCATAACAACAGCA	ACACCCTTCTCGTCCATGTC	155
β-1,3-glucanase	MD13G1181800	CCGTCCTTCTCGTCTCTGAC	CCGATTGCAGAAACACCTTT	143
Dehydrin	MD02G1139900	GGAGGAAGAAGAAGGGGTTG	TTGAGTAGTGGGGGTCATCC	186
PR1	MD05G1109100	CTTGACGTGGGATGACAATG	TACGCCAAACCACCTGTGTA	21
PR14	MD11G1054100	GGAGTGTTTGGAGGCACAGT	AGGACAAGGAGAGCCACAGA	186
MdTubulin		AGGATGCTACAGCCGATGAG	GCCGAAGAACTGACGAGAATC	192
