# Supplementary figures and images for: Transcriptome Analysis of Apple Leaves in Response to Powdery Mildew (Podosphaera leucotricha) Infection
Source: Int J Mol Sci. 2019 May 10;20(9):2326. doi: 10.3390/ijms20092326 (PMC6539105; doi:10.3390/ijms20092326)

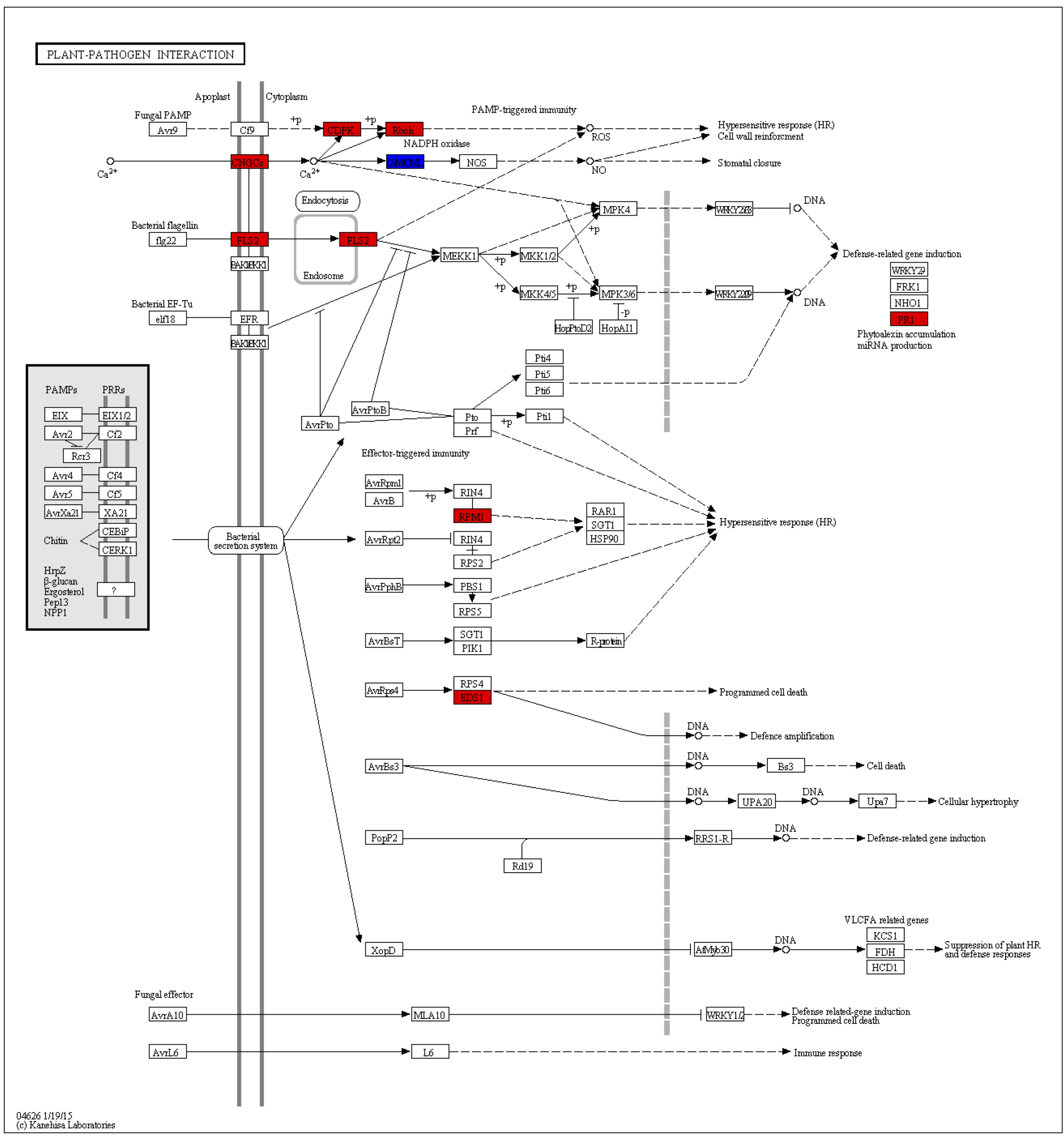

Supplement: Supplementary file 1 [file ijms-20-02326-s001.zip › All supplementary files/Figure S3. Plant pathogen interaction pathway map ko 04626.png]

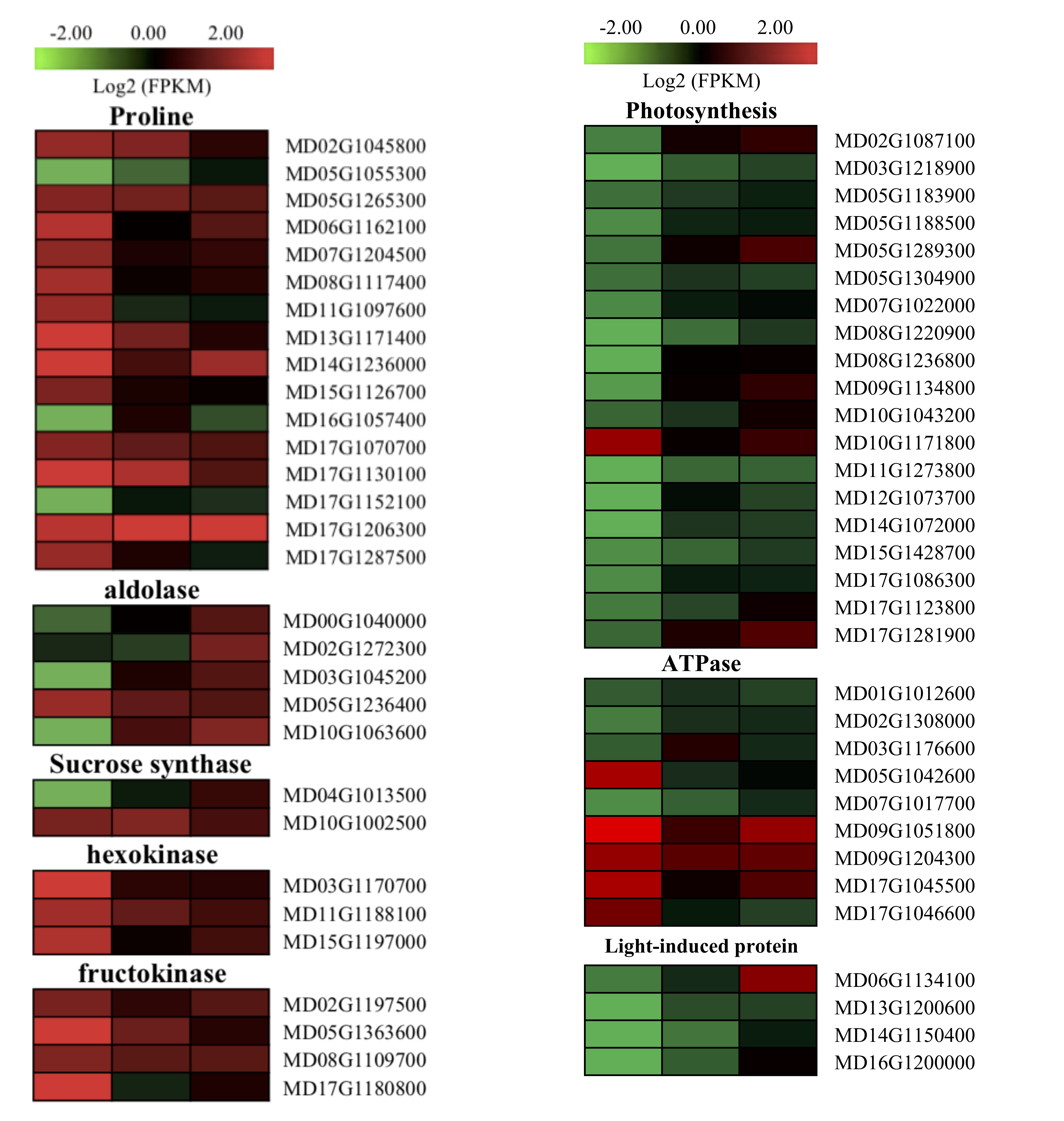

Supplement: Supplementary file 1 [file ijms-20-02326-s001.zip › All supplementary files/Figure S6..jpg]

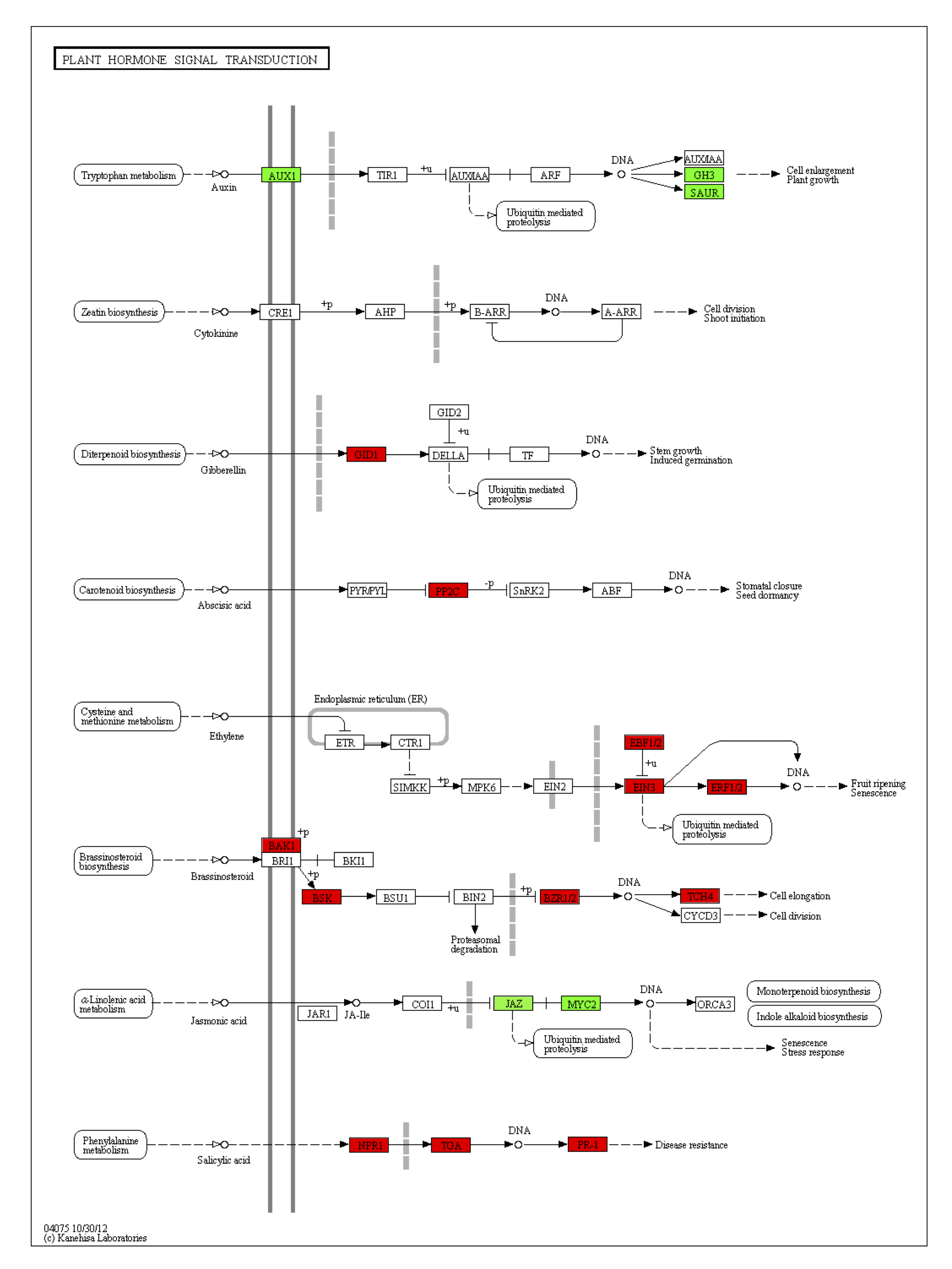

Supplement: Supplementary file 1 [file ijms-20-02326-s001.zip › All supplementary files/Figure S2. Plant hormone signal pathway map ko 04075.png]

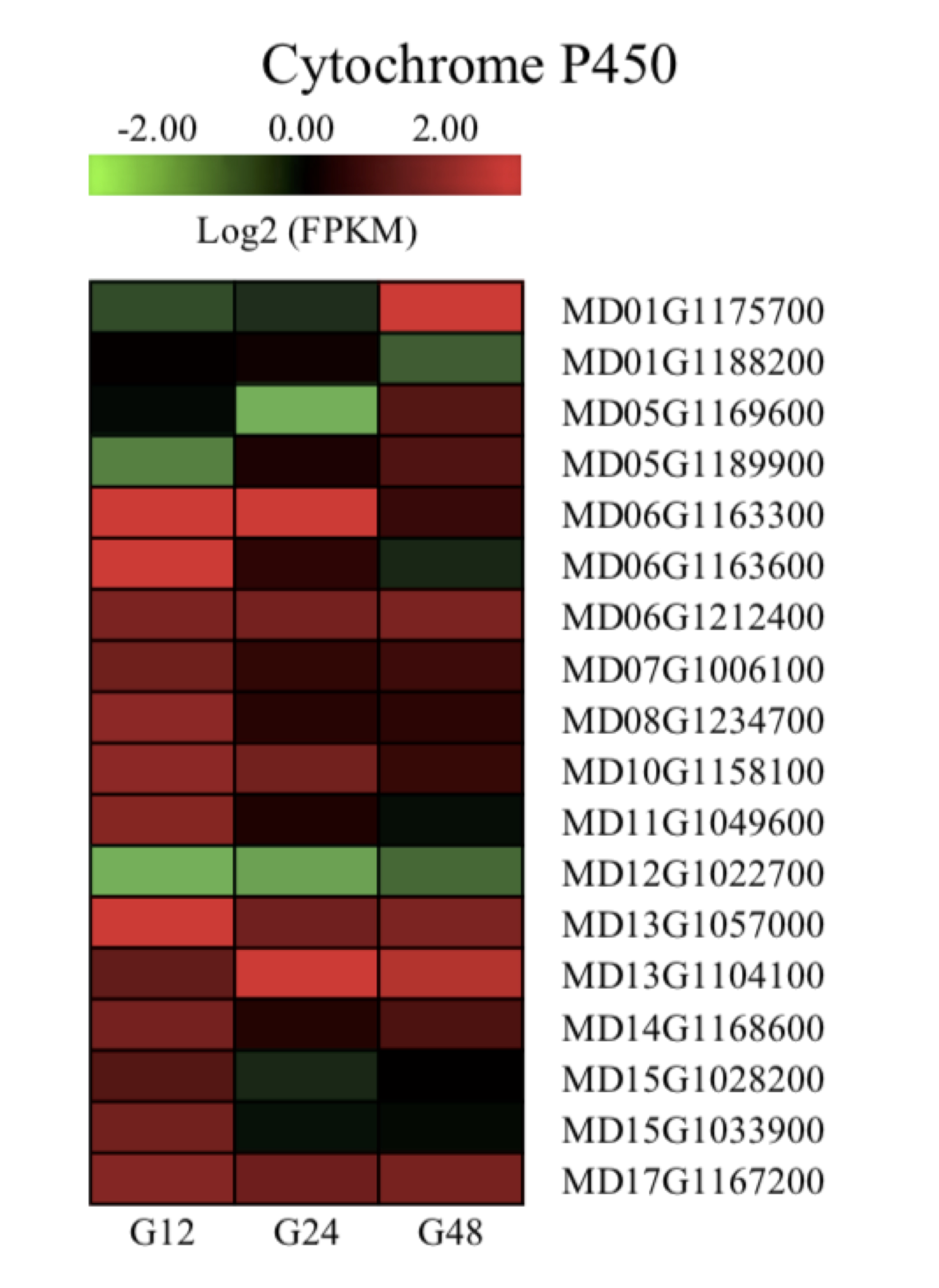

Supplement: Supplementary file 1 [file ijms-20-02326-s001.zip › All supplementary files/Figure S5.CYP.jpg]

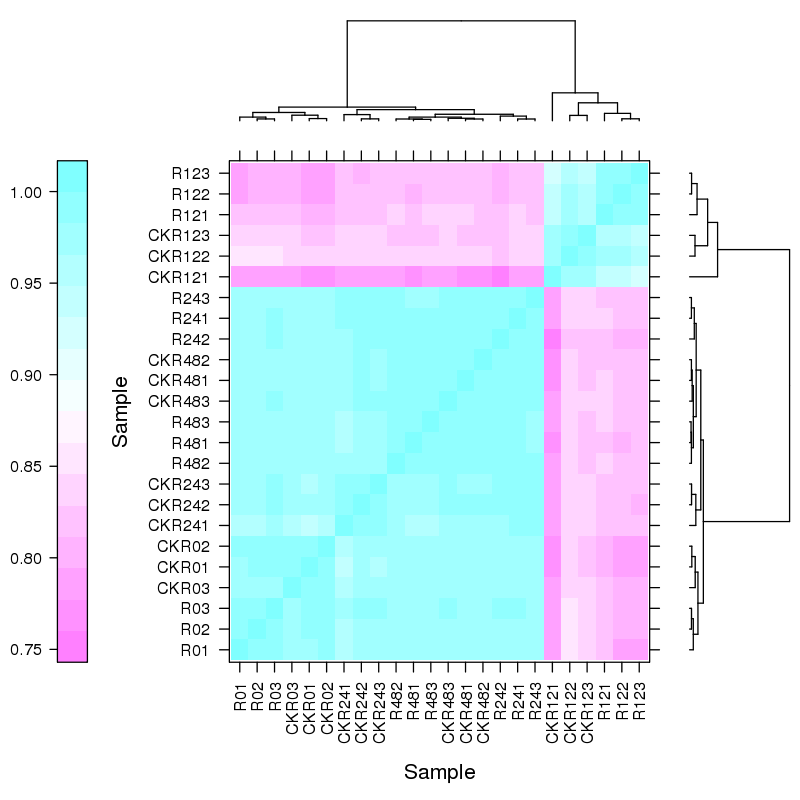

Supplement: Supplementary file 1 [file ijms-20-02326-s001.zip › All supplementary files/Figure S1.png]

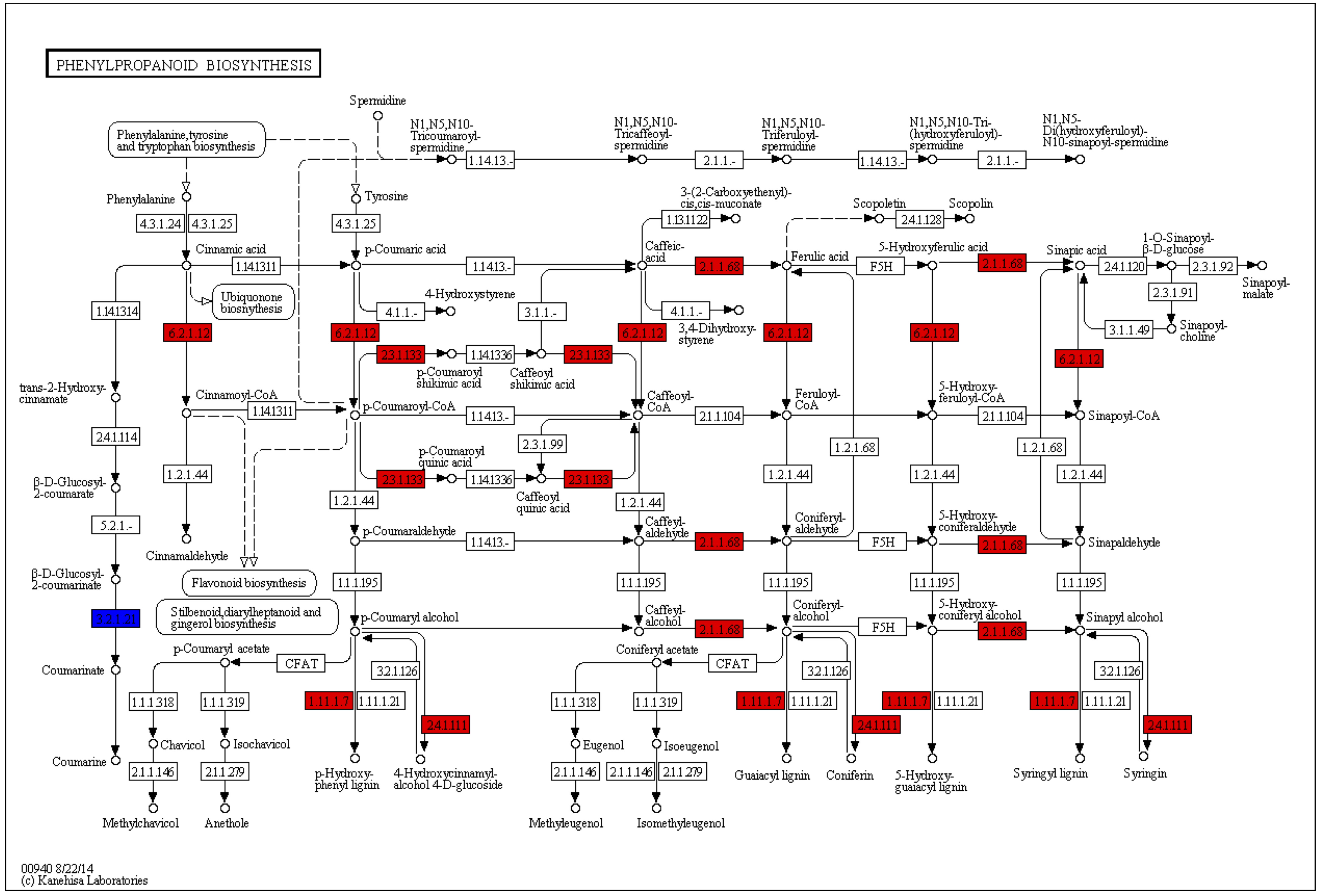

Supplement: Supplementary file 1 [file ijms-20-02326-s001.zip › All supplementary files/Figure S4. Phenylpropanoid biosynthesis pathway map ko 00940.png]
